# Complete Genome Sequence of Fowl Aviadenovirus Serotype 8b Isolated in South America

**DOI:** 10.1128/genomeA.01174-16

**Published:** 2016-10-20

**Authors:** Ray Izquierdo-Lara, Katherine Calderón, Ana Chumbe, Ricardo Montesinos, Ángela Montalván, Armando E. González, Eliana Icochea, Manolo Fernández-Díaz

**Affiliations:** a FARVET S.A.C., Chincha Alta, Ica, Peru; b Universidad Nacional Mayor de San Marcos, Faculty of Veterinary Medicine, San Borja, Lima, Peru

## Abstract

We present here the complete genome sequence of fowl aviadenovirus E (FAdV-E) serotype 8b strain FV211-16, isolated from chickens with inclusion body hepatitis in Peru. Genome comparisons with other FAdV-E strains revealed identities of 84.9 to 97.1% and the presence of 9 and 2 unique amino acid mutations in hexon and fiber proteins, respectively.

## GENOME ANNOUNCEMENT

Fowl aviadenoviruses (FAdVs) are worldwide-distributed viruses and are the causative agents of the inclusion body hepatitis (IBH) in chickens (1). IBH is characterized by hepatic necrosis with microscopic eosinophil or basophilic intranuclear inclusion bodies in hepatocytes and mortality rates around 10% (2). FAdVs have been grouped into five species (FAdV-A to FAdV-E) on the basis of their genome structure and further divided into 12 serotypes (FAdV-1 to -8a and -8b to -11), based on a cross-neutralization test (1). All serotypes have been associated with IBH outbreaks. Despite the presence of IBH outbreaks in South America (3–5), information about the circulating strains in this continent is still limited.

In January 2016, an IBH outbreak was observed in broiler chickens in a farm located in Arequipa, Peru. The outbreak was characterized by decreased weight gain, mortality rates around 5 to 10% in young chickens (2 to 4 weeks), and necrotic livers in affected birds. The virus was isolated from collected livers in LMH cells. Viral DNA was sequenced on the Genome Sequencer FLX platform (Roche, Mannheim, Germany). The *de novo* Assembler 2.6 software program was used for *de novo* assembly. Sequence alignment and phylogenetic analysis were performed on PRANK and Mega 6.0, respectively. An experimental intramuscular inoculation of FV211-16 in 21-day-old specific-pathogen-free (SPF) chickens, followed for 3 weeks, was performed under the approval of the Institutional Animal Care and Use Committee of the Universidad Nacional Mayor de San Marcos (UNMSM), Lima, Peru.

The full-genome sequence of FV211-16 was 43,976 nucleotides (nt) long, with 57.9% G+C content. The virus codifies 46 open reading frames (ORFs) and has a gene organization similar to that of FAdV-E (serotypes 6, 7, 8a, and 8b). Both inverted terminal repeat (ITR) sequences are 71 nt long and match perfectly to each other. Comparisons with the other six FAdV-E genomes available in the GenBank (as of July 2016) showed identities of 84.9 to 97.1%. Phylogenetic analyses based on complete genome sequences showed that FV211-16 clusters together with serotype 8b strains (accession numbers KU517714, GU734104, and KT862811), with identities between 95.4 and 97.1%, with the most closely related strain being the Malaysian strain UPM04217 (6). Amino acid alignments revealed that Penton protein was highly conserved through all FAdV-E strains, with identities between 97.8 and 100%. Compared with other FAdV-E strains, the hexon and fiber proteins of FV211-16 have nine and two unique amino acid mutations, respectively. The mutations in the hexon are mainly located in loop-1 and loop-2 regions, while mutations in fiber protein were located in positions 198 (shaft) and 396 (knob).

Under experimental conditions, FV211-16 (1.3 × 10^8^ PFU/bird) showed no mortality (0/20) and minimal clinical signs. Likewise, necropsy revealed mild gross lesions of IBH in the liver and intestinal tracks.

Overall, this is the first complete genome sequence of an FAdV circulating in South America. Moreover, the presence of FAdV-8b suggests that other strains may be circulating in our country. Surveillance should be increased to aid in the prevention of IBH.

### Accession number(s).

The complete genome sequence of FV211-16 has been deposited to GenBank under accession no. KX258422.
